# Exercise capacity and cardiac hemodynamic response in female ApoE/LDLR^−/−^ mice: a paradox of preserved V’O_2max_ and exercise capacity despite coronary atherosclerosis

**DOI:** 10.1038/srep24714

**Published:** 2016-04-25

**Authors:** M. Wojewoda, U. Tyrankiewicz, P. Gwozdz, T. Skorka, M. Jablonska, A. Orzylowska, K. Jasinski, A. Jasztal, K. Przyborowski, R. B. Kostogrys, J. A. Zoladz, S. Chlopicki

**Affiliations:** 1 Jagiellonian Centre for Experimental Therapeutics (JCET), Jagiellonian University, Krakow, Poland; 2 Department of Magnetic Resonance Imaging, Institute of Nuclear Physics, Polish Academy of Sciences Krakow, Poland; 3 Chair of Pharmacology, Jagiellonian University Medical College, Krakow, Poland; 4 Department of Human Nutrition, Faculty of Food Technology, University of Agriculture in Krakow, Krakow, Poland; 5 Department of Muscle Physiology, Faculty of Rehabilitation, University School of Physical Education, Krakow, Poland

## Abstract

We assessed exercise performance, coronary blood flow and cardiac reserve of female ApoE/LDLR^−/−^ mice with advanced atherosclerosis compared with age-matched, wild-type C57BL6/J mice. Exercise capacity was assessed as whole body maximal oxygen consumption (V’O_2max_), maximum running velocity (v_max_) and maximum distance (DIST_max_) during treadmill exercise. Cardiac systolic and diastolic function in basal conditions and in response to dobutamine (mimicking exercise-induced cardiac stress) were assessed by Magnetic Resonance Imaging (MRI) *in vivo*. Function of coronary circulation was assessed in isolated perfused hearts. In female ApoE/LDLR^−/−^ mice V’O_2max_, v_max_ and DIST_max_ were not impaired as compared with C57BL6/J mice. Cardiac function at rest and systolic and diastolic cardiac reserve were also preserved in female ApoE/LDLR^−/−^ mice as evidenced by preserved fractional area change and similar fall in systolic and end diastolic area after dobutamine. Moreover, endothelium-dependent responses of coronary circulation induced by bradykinin (Bk) and acetylcholine (ACh) were preserved, while endothelium-independent responses induced by NO-donors were augmented in female ApoE/LDLR^−/−^ mice. Basal COX-2-dependent production of 6-keto-PGF_1α_ was increased. Concluding, we suggest that robust compensatory mechanisms in coronary circulation involving PGI_2_- and NO-pathways may efficiently counterbalance coronary atherosclerosis-induced impairment in V’O_2max_ and exercise capacity.

Atherosclerosis is a major cause of death worldwide (for review see)^1^. It is linked to development of endothelial dysfunction, lipid retention in subendothelial space of arteries and vascular inflammation that leads to intravascular thrombosis and its clinical consequences such as coronary artery disease^2^. Atherosclerotic changes are not only present in disease states (i.e. coronary heart disease, peripheral arterial disease or type 2 diabetes), but also accompany physiological human aging and may be clinically silent for long periods of time^3^.

Atherosclerosis usually is associated with worse exercise capacity in men^4^,^5^,^6^. Furthermore, impaired exercise tolerance was reported to be a prognostic tool to predict the development of atherosclerotic changes in patients^7^,^8^,^9^. In single knockout ApoE^−/−^ mice, representing the most frequently used murine model of spontaneous atherosclerosis^10^, exercise capacity was also impaired compared to healthy mice^11^,^12^,^13^,^14^. Moreover, cardiac structure and function in ApoE^−/−^ mice was altered^15^,^16^, including reduction of their cardiac contractility reserve after dobutamine stimulation^17^. On the other hand, single knockout LDL^−/−^ mice covered distances comparable to their wild-type counterparts when accommodated in cages equipped with wheels that suggested their everyday exercise capacity was not compromised (please compare daily covered distances in Lightfoot *et al*.^18^ and O’Bryan *et al*.^19^).

Double ApoE^−/−^ and LDLR^−/−^ knockouts constitute a reliable model of human atherosclerosis that displays some advantages over single ApoE^−/−^ and LDLR^−/−^ knockouts^20^,^21^. Namely, in ApoE/LDLR^−/−^ mice plasma concentration of low density lipoprotein cholesterol fraction is very high, and spontaneous atherosclerosis develops more rapidly compared to ApoE^−/−^ mice. Moreover, a western diet is not needed to induce atherosclerosis as in LDLR^−/−^ mice. Furthermore, arterial plaques become advanced atherosclerotic plaques earlier and resemble those observed in humans^22^. Surprisingly, even though ApoE/LDLR^−/−^ mice develop intensive coronary atherosclerosis and some authors report higher susceptibility of their hearts to hypoxic stress^23^,^24^, no reports published so far provide evidence of impaired cardiac function in ApoE/LDLR^−/−^ mice^25^. Furthermore, there are no data in the literature regarding exercise capacity and cardiac haemodynamic response to exercise in ApoE/LDLR^−/−^ mice. Therefore, we examined whole body maximal oxygen uptake (V’O_2max_) and exercise running capacity, as well as NO- and PGI_2_-dependent function of the coronary circulation of female ApoE/LDLR^−/−^ vs C57BL6/J mice in relationship to atherosclerosis development. We also measured basal cardiac function and dobutamine-stimulated cardiac performance that mimics exercise-induced haemodynamic changes in 6–8-month old female ApoE/LDLR^−/−^ mice with advanced atherosclerosis as compared with age-matched C57BL6/J mice.

## Results

### Exercise capacity of female ApoE/LDLR^−/−^ and C57BL6/J mice

To measure effects of atherosclerosis development on exercise capacity of female ApoE/LDLR^−/−^ mice we measured whole body maximal oxygen consumption (V’O_2max_), maximal velocity (v_max_) achieved and maximal distance (DIST_max_) covered during incremental test for V’O_2max_ measurement by young 3-month old female ApoE/LDLR^−/−^ mice with early atherosclerosis and older 6–8-month old female ApoE/LDLR^−/−^ mice with advanced atherosclerosis^26^ as compared with age-matched control C57BL6/J mice.

**Figure 1:**
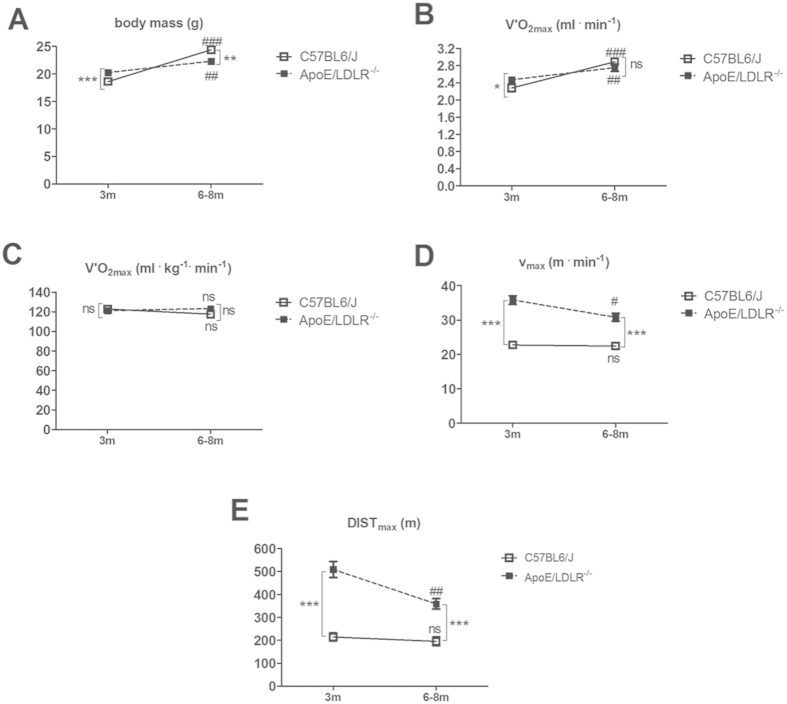
Running performance in female ApoE/LDLR^−/−^ mice and C57BL6/J mice. (**A**) Body mass, (**B**,**C**) whole body maximal oxygen consumption (V’O_2max_) during incremental test, (**D**) maximal velocity (v_max_) and (**E**) maximal distance (DIST_max_) in running incremental test in 3-month (3m) and 6–8-month (6-8m) old ApoE/LDLR^−/−^ and C57BL6/J mice. Statistical analysis was performed using two-sided t-test or non-parametric Mann-Whitney test depending on the results of D’Agostino and Pearson omnibus normality test. *indicates statistically significant difference between C57BL6/J and ApoE/LDLR^−/−^ mice, ^#^indicates statistically significant age-dependent difference within the strain. ^*,#^P < 0.05, ^##^P < 0.01, ^***,###^P < 0.001; n = 16–20 for 3-month old C57BL6/J mice, n = 16–19 for 3-month old ApoE/LDLR^−/−^ mice, n = 9–11 for 6–8-month old C57BL6/J mice and n = 11 for 6–8-month old ApoE/LDLR^−/−^ mice. Data are presented as the mean ± SEM.

Body mass of young female ApoE/LDLR^−/−^ mice was higher than control C57BL6/J mice while for older female ApoE/LDLR^−/−^ and C57BL6/J mice we found the opposite (Fig. 1A) and it increased with age in both mouse strains. Therefore, the data regarding V’O_2max_ are presented both in absolute (Fig. 1B) and relative values (Fig. 1C). There was no difference in V’O_2max_ expressed in relative values between the mouse strains (Fig. 1C). However, when expressed in absolute values, changes in V’O_2max_ reflected changes in body weight (please compare Fig. 1A,B).

Surprisingly, maximal velocities (v_max_) achieved by female ApoE/LDLR^−/−^ were higher than for age-matched C57BL6/J mice (35.84 m^·^min^−1^ vs 22.8 ± 0.84 m^·^min^−1^, n = 19–20, P < 0.0001 for younger female ApoE/LDLR^−/−^ and C57BL6/J mice, respectively, and 30.82 ± 1.12 m^·^min^−1^ vs 22.45 ± 0.97 m^·^min^−1^, n = 11, P < 0.0001 for older female ApoE/LDLR^−/−^ and C57BL6/J mice, respectively) (Fig. 1D). Similarly, maximal distances (DIST_max_) covered by female ApoE/LDLR^−/−^ and C57BL6/J mice were higher for female ApoE/LDLR^−/−^ mice than for age-matched C57BL6/J mice (509.2 ± 34.97 m vs 214.8 ± 0.8417.15 m, n = 19–20, P < 0.0001 for younger female ApoE/LDLR^−/−^ and C57BL6/J mice, respectively, and 359.4 ± 22.9 m vs 195.9 ± 19.04 m, n = 11, P < 0.0001 for older female ApoE/LDLR^−/−^ and C57BL6/J mice, respectively) (Fig. 1E). Interestingly, v_max_ and DIST_max_ declined with age only in female ApoE/LDLR^−/−^ mice but not in C57BL6/J mice (35.84 m^·^min^−1^ for younger vs 30.82 ± 1.12 m^·^min^−1^ for older female ApoE/LDLR^−/−^ mice, n = 11−19, P = 0.0118 and 509.2 ± 34.97 m for younger vs 359.4 ± 22.9 m for older female ApoE/LDLR^−/−^ mice, n = 11, P = 0.0052, respectively) probably due to atherosclerosis progression (Fig. 1D,E).

Measurement of blood count revealed that both younger and older female ApoE/LDLR^−/−^ mice had higher number of circulating erythrocytes when compared with age-matched C57BL6/J mice (Table 1). However, number of erythrocytes declined with age in female ApoE/LDLR^−/−^ mice what corresponded with age-dependent decline in v_max_ and DIST_max_ covered by female ApoE/LDLR^−/−^ mice in the incremental test for V’O_2max_ measurement (Fig. 1D,E).

### NO- and PGI_2_-dependent function of coronary circulation in female ApoE/LDLR^−/−^ and C57BL6/J mice

**Figure 2:**
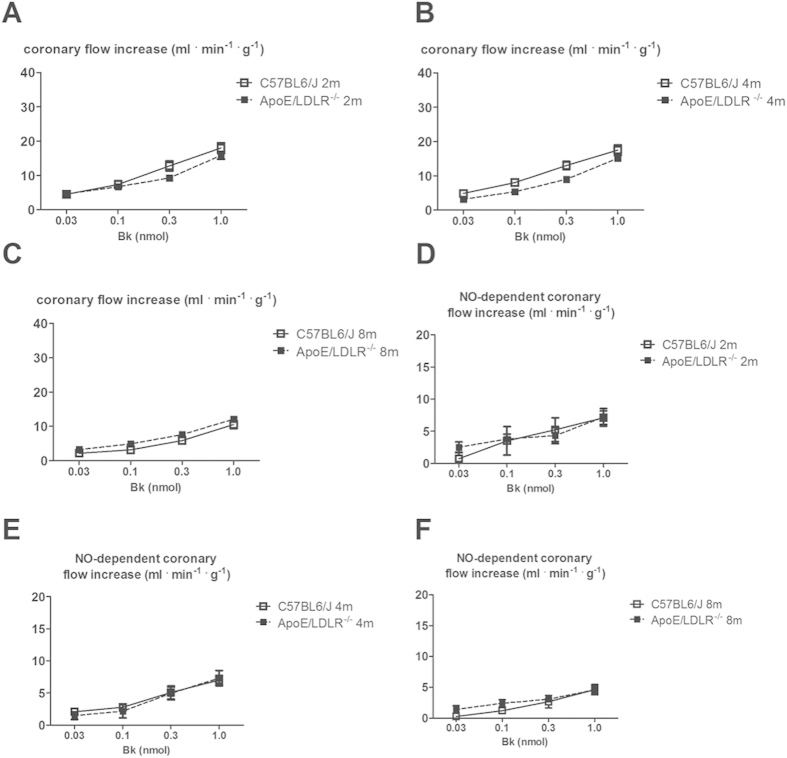
Bk–induced vasodilation responses in the coronary circulation of isolated heart in female ApoE/LDLR^−/−^ mice. (**A**–**C**) Preservation of coronary flow responses evoked by Bk in the isolated hearts of 2, 4 and 8-month old ApoE/LDLR^−/−^ mice compared with age-matched C57BL6/J mice; n = 11 for 2-month old C57BL6/J mice, n = 13 for 2-month old ApoE/LDLR^−/−^ mice, n = 14 for 4-month old C57BL6/J mice and n = 10 for 4-month old ApoE/LDLR^−/−^ mice, n = 9 for 8-month old C57BL6/J mice and n = 18 for 8-month old ApoE/LDLR^−/−^ mice. (**D**–**F**) NO-dependent part of coronary flow responses evoked by Bk in the isolated heart of ApoE/LDLR^−/−^ mice compared with controls subjected to L-NAME (500 μM) treatment; n = 6 for 2-month old C57BL6/J mice, n = 6 for 2-month old ApoE/LDLR^−/−^ mice, n = 7 for 4-month old C57BL6/J mice and n = 8 for 4-month old ApoE/LDLR^−/−^ mice, n = 5 for 8-month old C57BL6/J mice and n = 8 for 8-month old ApoE/LDLR^−/−^ mice. Statistical analysis was performed using t-test or non-parametric Mann-Whitney test, when assumptions were not completed. Data are presented as the mean ± SEM.

**Figure 3:**
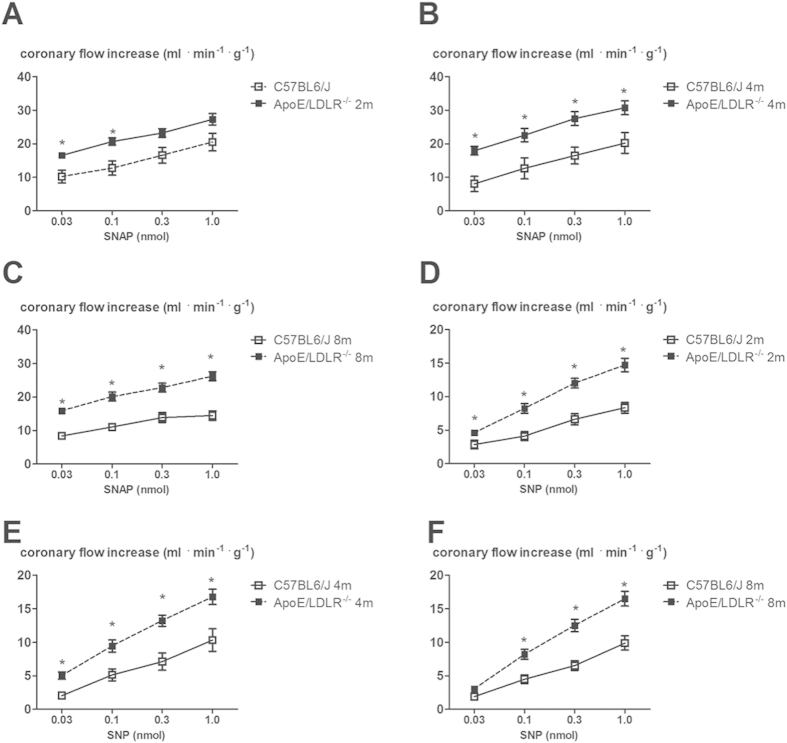
SNAP and SNP–induced vasodilation responses in the coronary circulation of isolated heart in female ApoE/LDLR^−/−^ mice. (**A**–**C**) Up-regulation of SNAP-induced coronary flow response in the isolated hearts of in 2, 4 and 8-month old ApoE/LDLR^−/−^ mice compared with age-matched C57BL6/J mice; n = 12 for 2-month old C57BL6/J mice, n = 7 for 2-month old ApoE/LDLR^−/−^ mice, n = 6 for 4-month old C57BL6/J mice and n = 9 for 4-month old ApoE/LDLR^−/−^ mice, n = 7 for 8-month old C57BL6/J mice and n = 13 for 8-month old ApoE/LDLR^−/−^ mice. (**D**–**F**) SNP-induced coronary flow response in isolated hearts from ApoE/LDLR^−/−^ mice compared with age-matched C57BL6/J mice; n = 7 for 2-month old C57BL6/J mice, n = 14 for 2-month old ApoE/LDLR^−/−^ mice, n = 5 for 4-month old C57BL6/J mice and n = 14 for 4-month old ApoE/LDLR^−/−^ mice, n = 10 for 8-month old C57BL6/J mice and n = 15 for 8-month old ApoE/LDLR^−/−^ mice. Statistical analysis was performed using t-test or non-parametric Mann-Whitney test, when assumptions were not completed. Data are presented as the mean ± SEM.

We previously showed that in mice, in contrast to humans^27^ and guinea pigs^28^, acetylcholine-induced response in coronary circulation was mediated mainly by prostacyclin (PGI_2_)^29^ whereas Bk-induced response was mediated to a large extent by NO. In this study we found that, in spite of age-dependent peripheral atherosclerosis progression described earlier^26^ and coronary atherosclerosis progression described in this paper, changes in coronary flow induced by Bk (0.03, 0.1, 0.3, 1.0 nmol) in isolated hearts from female ApoE/LDLR^−/−^ mice were not impaired compared with age-matched C57BL6/J mice (Fig. 2A–C). Moreover, the inhibitory effect of L-NAME on the changes in coronary flow after Bk in C57BL6/J and in female ApoE/LDLR^−/−^ mice hearts was similar (Fig. 2D–F). Interestingly, SNAP and SNP-induced coronary flow responses were augmented in 2, 4 and 8-month old female ApoE/LDLR^−/−^ mice compared with age-matched C57BL6/J mice (Fig. 3).

**Figure 4:**
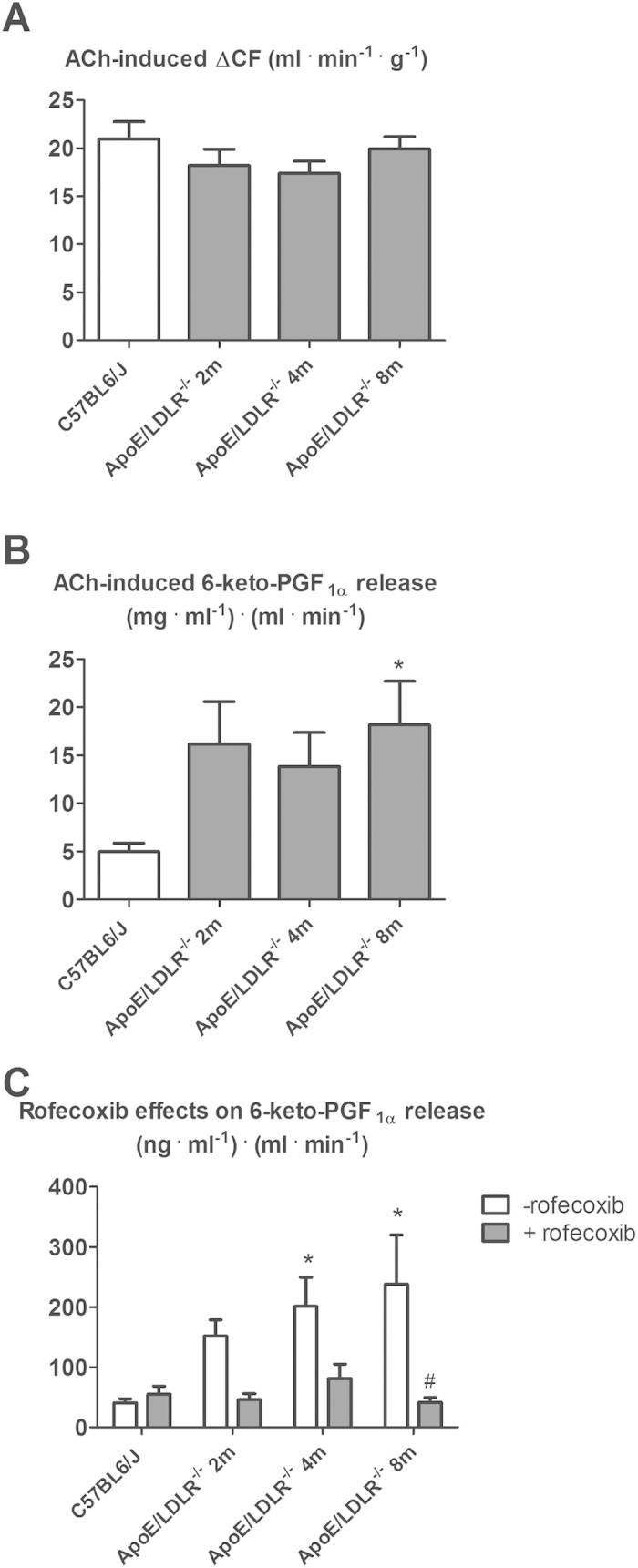
PGI_2_-dependent coronary vasodilation in the coronary circulation of isolated heart in female ApoE/LDLR^−/−^ mice. (**A**) PGI_2_-dependent coronary flow response evoked by ACh (0.3 nmol) in isolated hearts of ApoE/LDLR^−/−^ mice compared with control C57BL6/J mice. Since there was no statistically significant difference in Ach—induced changes in coronary blood flow between 2, 4 and 8-month old C57BL6/J mice, the results for C57BL6/J mice were averaged; n = 25 for C57BL6/J mice, n = 23 for 2-month old ApoE/LDLR^−/−^ mice, n = 16 for 4-month old ApoE/LDLR^−/−^ mice, n = 22 for 8-month old ApoE/LDLR^−/−^ mice. (**B**) ACh-induced 6-keto-PGF_1α_ release in isolated heart from ApoE/LDLR^−/−^ mice compared with age-matched C57BL6/J mice. Since there was no statistically significant difference in Ach—induced changes of 6-keto-PGF_1α_ release between 2, 4 and 8-month old C57BL6/J mice, the results for C57BL6/J mice were averaged; n = 15 for C57BL6/J mice, n = 7 for 2-month old ApoE/LDLR^−/−^ mice, n = 9 for 4-month old ApoE/LDLR^−/−^ mice, n = 11 for 8-month old ApoE/LDLR^−/−^ mice. (**C**) Effects of COX-2 inhibitor (rofecoxib, 10 μM) on basal 6-keto-PGF_1α_ production in the isolated heart of ApoE/LDLR^−/−^ mice. Since there was no statistically significant difference in changes of 6-keto-PGF_1α_ release between 2, 4 and 8-month old C57BL6/J mice (basal and after rofecoxib treatment), the results for C57BL6/J mice were averaged; n = 11 and 5 for C57BL6/J mice, n = 8 and 6 for 2-month old ApoE/LDLR^−/−^ mice, n = 9 and 6 for 4-month old ApoE/LDLR^−/−^ mice, n = 9 and 6 for 8-month old ApoE/LDLR^−/−^ mice with respect to basal and after-rofecoxib 6-keto-PGF_1α_ release. Statistical analysis was performed using t-test or non-parametric Mann-Whitney test (if the assumptions were not completed). *indicates P < 0.05 for ApoE/LDLR^−/−^ vs. control mice. ^#^indicates P < 0.05 with rofecoxib vs. without. Data are presented as the mean ± SEM.

ACh-induced coronary flow response in 2, 4 and 8-month old female ApoE/LDLR^−/−^ mice was not impaired (Fig. 4A), but ACh-induced 6-keto-PGF_1α_ release into cardiac effluent increased (18.18 ± 12.58 mg^·^ml^−1^ ^.^ml^·^min^−1^ for 8-month old female ApoE/LDLR^−/−^ mice, n = 11, compared to 4.99 ± 2.65 mg^·^ml^−1^^.^ml^·^min^−1^ for controls, n = 15, P < 0.05, Fig. 4B). It is worth to note that 6-keto-PGF_1α_ release into cardiac effluent tended to be higher also for 2 and 4-month old female ApoE/LDLR^−/−^ mice compared with control mice but the difference did not reach statistical significance. Basal 6-keto-PGF_1α_ production in the hearts of female ApoE/LDLR^−/−^ mice was also increased (238.6 ± 193.10 mg^·^ml^−1^^.^ml^·^min^−1^ for 8 months old female ApoE/LDLR^−/−^ mice, n = 9, compared to 41.3 ± 17.02 mg^·^ml^−1·^ml^·^min^−1^ for controls, n = 11, P < 0.05) (Fig. 4C) and the difference in PGI_2_ production between female ApoE/LDLR^−/−^ and C57BL6/J mice was abolished by COX-2 selective inhibitor, rofecoxib.

Since there was no statistically significant difference in Ach—induced changes in coronary flow, Ach—induced changes in 6-keto-PGF_1α_ release and changes in 6-keto-PGF_1α_ release (basal and after rofecoxib treatment) between 2, 4 and 8-month old C57BL6/J mice, the results for C57BL6/J mice were averaged.

### Atherosclerotic lesions in coronary arteries of female ApoE/LDLR^−/−^ mice

**Figure 5:**
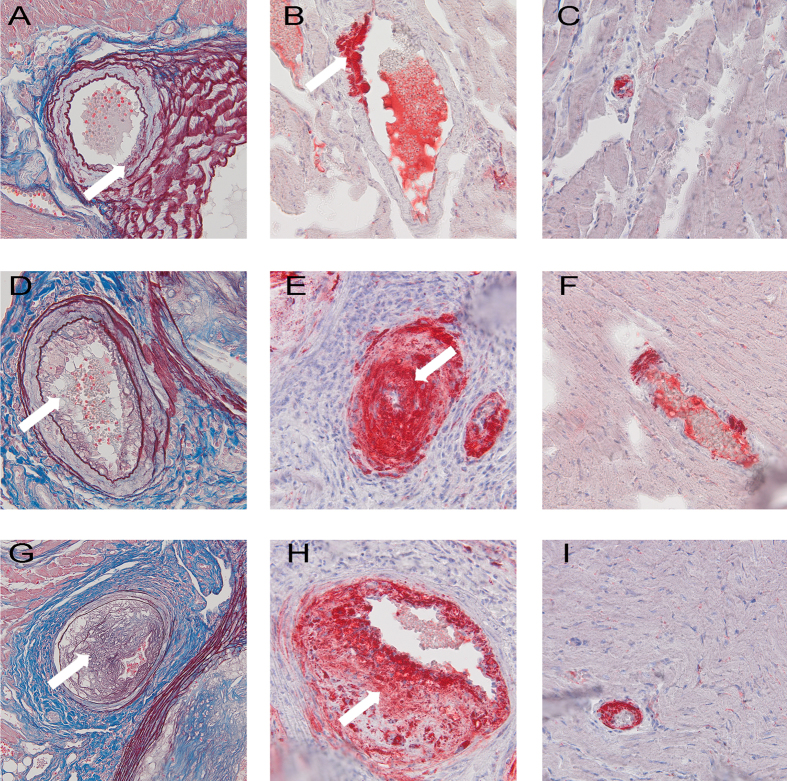
Coronary atherosclerosis in coronary arteries of female ApoE/LDLR^−/−^ mice. Large coronary arteries with atherosclerotic plaques in 2-month old (**A**), 4-month old (**D**) and 6–8-month old (**G**) female ApoE/LDLR^−/−^ mice and their lipid components ((**B**,**E**,**H**), respectively); small atherosclerotic lesions are only occasionally found in large coronary arteries of 2-month old ApoE/LDLR^−/−^ mice (**A**,**B**) but become evident in older mice (**D**,**E**,**G**,**H**) (white arrows). The lumen of small intramuscular coronary arteries of 2, 4 and 6–8month old ApoE/LDLR^−/−^ mice ((**C**,**F**,**I**), respectively) is almost completely occluded by atherosclerotic plaque. OMSB staining was used to depict the morphology of atherosclerotic plaques (**A**,**D**,**G**) and ORO was used to visualise significant lipid deposits within the plaques (**B**,**C**,**E**,**F**,**H**,**I**). Magnification 400×.

Histological analysis of atherosclerotic plaques in coronary circulation of 2, 4 and 6–8 month old female ApoE/LDLR^−/−^ mice revealed that in initial parts of large coronary arteries branching from the aorta (proximal) age-related development of atherosclerotic lesions was found (A and B, D and E, G and H, respectively) whereas small intramuscular coronary arteries (distal) (Fig. 5C,F,I) displayed advanced atherosclerotic lesions largely occluding the vessel lumen. The middle parts of the coronary arteries were less affected (results not shown).

### Cardiac performance at rest and under dobutamine stimulation in female ApoE/LDLR^−/−^ and C57BL6/J mice

**Figure 6:**
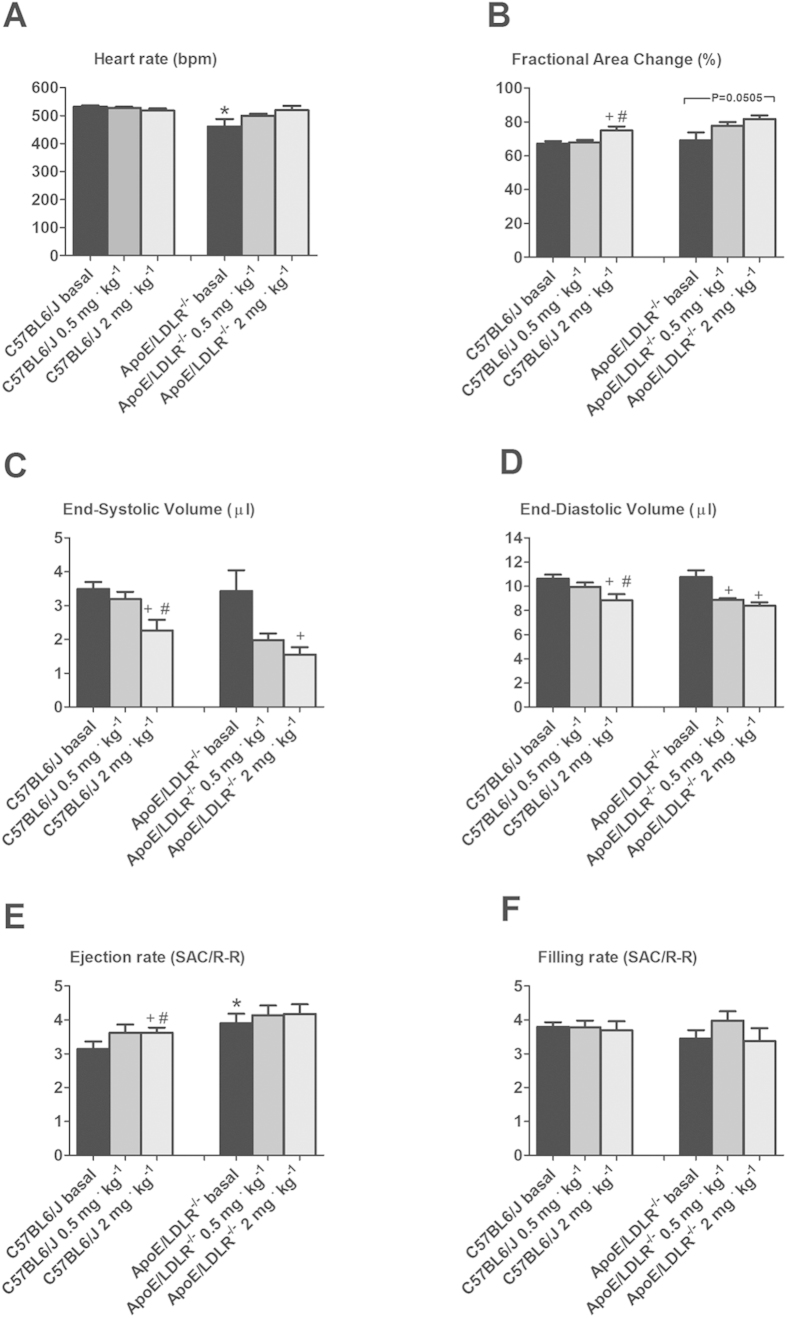
Cardiac reserve for low (0.5 mg^·^kg^−1^) and high (2.0 mg^·^kg^−1^) dose of dobutamine in female ApoE/LDLR^−/−^ mice. Dobutamine stress test uncovered well-preserved cardiac reserve in ApoE/LDLR^−/−^ mice. *indicates statistical significance in comparison of the ApoE/LDLR^−/−^ and the control mice at basal conditions (P < 0.05, t-test; n = 7 in each group), whereas changes after dobutamine were marked as + for comparisons between the given dose of dobutamine (low or high) and the basal condition, and as # for comparisons between the high and low dose (P < 0.05, ANOVA and Tukey *post-hoc* test); n = 7–8 for C57BL6/J mice and 6 for ApoE/LDLR^−/−^ mice. Data are presented as the mean ± SEM.

Basal left ventricle function including fractional area change (FAC), end systolic and end diastolic volume (ESV, EDV) and filling rate (FR) were not changed in 6–8-month old female ApoE/LDLR^−/−^ mice compared to age-matched C57BL6/J mice (P < 0.05, Fig. 6B,C,D,F). The only difference was that the heart rate was accelerated in C57BL6/J mice and basal ejection rate was higher in female ApoE/LDLR^−/−^ mice (P < 0.05, Fig. 6A,E, respectively). Not only was basal cardiac function not compromised in female ApoE/LDLR^−/−^ mice as compared to age-matched C57BL6/J mice but also response to dobutamine stress was fully preserved. As shown in Fig. 6, dobutamine stimulation at low (0.5 mg^·^kg^−1^) and high dose (2 mg^·^kg^−1^) demonstrated fully preserved, inotropic and lusitropic response in female ApoE/LDLR^−/−^ mice. In fact, the increase in FAC (from basal 0.67 ± 0.03 to 0.75 ± 0.06 and from 0.68 ± 0.11 to 0.82 ± 0.04 for controls vs. female ApoE/LDLR^−/−^ mice respectively, P < 0.05, Fig. 6B–D) and decrease in ESA (from 3.49 ± 0.53 to 2.27 ± 0.81 and from 3.43±1.51 to 1.55 ± 0.53 for controls vs. female ApoE/LDLR^−/−^ mice respectively, P < 0.05) and EDA (from 10.63 ± 0.9 to 8.85 ± 1.31 and from 10.75 ± 1.38 to 8.39 ± 0.67 for controls vs. female ApoE/LDLR^−/−^ mice respectively, P < 0.05) were all comparable for female ApoE/LDLR^−/−^ and age-matched C57BL6/J mice. In contrast, chronotropic reserve was observed only in female ApoE/LDLR^−/−^ mice (P < 0.05, not significantly, Fig. 6A). No reserve in ejection rate was observed in female ApoE/LDLR^−/−^ mice, however their basal ejection rate was higher than in C57BL6/J mice (3.91 ± 0.68 for female ApoE/LDLR^−/−^ mice as regards 3.35 ± 0.46 for controls, P < 0.05, Fig. 6E). Both groups responded similarly as regards filling rate showing no changes under dobutamine stress (P < 0.05, Fig. 6F).

## Discussion

In this study we characterized whole body maximal oxygen uptake (V’O_2max_), exercise capacity and NO- and PGI_2_-dependent coronary endothelial function of female ApoE/LDLR^−/−^ mice with respect to age-related peripheral atherosclerosis progression described elsewhere^26^ and age-related coronary atherosclerosis progression shown here. We also characterized cardiac haemodynamic response to dobutamine-induced stress in 6–8 month old female ApoE/LDLR^−/−^ mice with advanced coronary atherosclerosis in comparison with age-matched C57BL6/J mice. Surprisingly, we found that V’O_2max_ considered a key index of cardio-respiratory capacity and running exercise capacity of female ApoE/LDLR^−/−^ mice were preserved irrespective of atherosclerosis progression (Fig. 1). Moreover, both younger (3-month old) female ApoE/LDLR^−/−^ mice and older (6–8-month old) female ApoE/LDLR^−/−^ mice with early and advanced peripheral (as reported earlier^26^) and early and advanced coronary (as shown here) atherosclerosis achieved higher velocities and covered longer distances than age-matched C57BL6/J mice (Fig. 1D,E). Excellent running exercise capacity of female ApoE/LDLR^−/−^ mice was associated with preserved NO-dependent function of coronary circulation (Fig. 2) and increased COX-2-dependent PGI_2_ production (Fig. 4C). Moreover, cardiac performance at rest as well as its inotropic and lusitropic reserve were well preserved in 6–8-month old female ApoE/LDLR^−/−^ mice (Fig. 6) with advanced coronary atherosclerosis (Fig. 5G–I) compared with age-matched C57BL6/J mice as evidenced by similar FAC, and slice-derived ESV and EDV in basal conditions as well as in response to dobutamine. Altogether, the present work demonstrated that development of atherosclerosis did not compromise V’O_2max_ of female ApoE/LDLR^−/−^ mice and did not influence their cardiac function even at the advanced stage of atherosclerosis with advanced atherosclerotic plaques in the coronary circulation and even during stress conditions. Running exercise capacity of 6–8-month old female ApoE/LDLR^−/−^ mice was only slightly lower than in young female ApoE/LDLR^−/−^ mice (Fig. 1D,E). Nevertheless, it was still better than in age-matched C57BL6/J mice. We claim that robust compensatory mechanisms in coronary circulation, including increased vascular responsiveness to NO (Fig. 3) and increased generation of PGI_2_ (Fig. 4B,C) that are triggered in the early phase of atherosclerosis development, could, at least partly, account for preserved V’O_2max_ and running exercise capacity of female ApoE/LDLR^−/−^ mice as well as their full cardiac adaptation to exercise occurring even at the stage of advanced atherosclerosis at the age of 6–8 months.

Given that it is widely known that atherosclerosis progression severely impairs cardiac reserve and exercise capacity in humans^4^,^5^,^6^ and in atherosclerotic ApoE^−/−^ mice of both genders^11^,^13^,^14^,^17^, our results regarding V’O_2max_, running exercise capacity and cardiac function of female ApoE/LDLR^−/−^ mice are surprising. Indeed, exercise capacity of female ApoE/LDLR^−/−^ mice was preserved despite peripheral (as reported previously^26^) and coronary (Fig. 5) atherosclerosis progression what could be a factor that could limit their exercise capacity^30^. These results can be only explained by important compensatory mechanisms triggered by double knockout of ApoE and Ldlr genes that preserved cardiac haemodynamic response to exercise of female ApoE/LDLR^−/−^ mice (Fig. 6) and assured sufficient adaptation of blood flow and oxygen delivery to their cardiac muscle during exercise which is the major determinant of V’O_2max_ (for review see^31^). These compensatory mechanisms could be, at least partly, associated with estrogens known to exert cardioprotective and vasoprotective effects. In fact, it was shown that in female atherosclerotic LDLR^−/−^ mice estrogens up-regulate cyclooxygenase 2 (COX-2) –derived production of PGI_2_ via estrogen receptor subtype alpha^32^.

It is also worth noting that oxygen extraction ratio within coronary circulation is already nearly maximal at rest and, thus, the major mechanism involved in increasing oxygen delivery during exercise is based on coronary vasodilation (and increased coronary blood flow), while in the skeletal muscles an increase in oxygen delivery to working muscles is brought about by an increase in its extraction ratio and recruitment of capillaries, as well as increased blood flow^31^. Considering this, it is the coronary and but not the skeletal muscle circulation that represents a *locus minoris resistantiae* in the regulation of V’O_2max_ and exercise capacity. Accordingly, compensatory mechanisms aimed to preserve V’O_2max_ in female ApoE/LDLR^−/−^ mice should be, first of all, present within the coronary circulation to preserve cardiac haemodynamic response to exercise. Indeed, our results fully support this assumption. In line with fully preserved basal cardiac function and stress response to dobutamine (Fig. 6), NO- and PGI_2_-dependent function of coronary circulation in female ApoE/LDLR^−/−^ mice was not compromised (Figs 2,3 and 4). On the contrary, clear-cut compensatory mechanisms in these mice were detected such as increased COX-2-dependent basal PGI_2_ production, increased COX-1-dependent PGI_2_ release in response to ACh as well as increased coronary vessel responsiveness to exogenous NO most likely linked to upregulation of soluble guanylate cyclase (sGC) activity in coronary vessels. Interestingly, these mechanisms were already evident in hearts of female ApoE/LDLR^−/−^ mice as early as at two months of age, suggesting that these compensatory mechanisms of coronary circulation are triggered before peripheral atherosclerotic plaques development^26^ and are probably induced by early coronary atherosclerosis (Fig. 5A–C). Considering vasoprotective and anti-atherosclerotic activity of NO-sGC and COX-2-PGI_2_ pathways, it is tempting to speculate that these compensatory changes within coronary circulation could mitigate the development of symptomatic ischaemic heart disease in female ApoE/LDLR^−/−^ mice and explain why their cardiac function was not compromised (Fig. 6) despite extensive coronary atherosclerosis at the age of 6–8 months (Fig. 5G–I).

To counteract detrimental results of peripheral endothelial dysfunction in female ApoE/LDLR^−/−^ mice, compensatory mechanisms induced by atherosclerosis also developed within peripheral circulation^26^. Namely, the cyclooxygenase-2 (COX-2)/PGI_2_ pathway was upregulated in the aorta of female ApoE/LDLR^−/−^ mice as early as at two months of age and remained elevated in older animals. Although we did not analyse endothelium-dependent regulation of blood flow in skeletal muscles, it could well be that exercise-induced increase in blood flow in skeletal muscles was also preserved due to compensatory mechanisms triggered by NO-deficiency in female ApoE/LDLR^−/−^ mice.

The mechanisms controlling coronary and muscle blood flow during exercise are not entirely understood and involve multiple endothelium-dependent vasodilatory pathways compensating one another. NO, PGI_2_ and RBC-derived ATP were claimed to be the major mediators involved^31^. It is widely known that NO is an important coronary and peripheral vasodilator during exercise^33^. However, in view of the available data, NO action alone could not explain the huge exercise-induced increase in skeletal muscles^34^ and coronary blood flow^35^. There is growing body of evidence that exercise-induced PGI_2_ might play an important role in regulation of coronary and muscle blood flow. It was demonstrated that the magnitude of exercise-induced PGI_2_ release correlated positively with V’O_2max_ in healthy humans^36^, while plasma nitrite concentration did not increase even upon vigorous exercise (for review see)^31^. Moreover, training-induced increase in V’O_2max_ was also associated with increased exercise-induced PGI_2_ release^37^. Furthermore, administration of PGI_2_ to patients with pulmonary hypertension increased their exercise capacity^38^. Extrapolating from human studies, increased PGI_2_ release into coronary (Fig. 4C) and peripheral^26^ circulation of female ApoE/LDLR^−/−^ mice could account for their preserved V’O_2max_ (Fig. 1B,C) and, paradoxically better running exercise capacity (Fig. 1D,E) as well as their preserved basal and dobutamine-stimulated cardiac function (Fig. 6).

Interestingly, there was also a higher number of circulating erythrocytes in female ApoE/LDLR^−/−^ mice compared with age-matched C57BL6/J control mice (Table 1). This could represent yet another mechanism that assured adequate oxygen supply to working tissues. Apart from oxygen transport, haemoglobin inside red blood cells reduces plasma nitrite (NO_2_^−^) to NO in hypoxic conditions that is subsequently released and contributes to local vasodilation^31^,^39^,^40^,^41^. When oxygen tension decreases within coronary microvasculature, red blood cells also release ATP which is a factor increasing coronary blood flow during exercise^42^. Increased number of circulating erythrocytes in female ApoE/LDLR^−/−^ mice points to increased erythropoesis in these animals (probably to counteract hypoxia). Indeed, erythropoietin production was shown to be higher in mice with atherosclerosis^43^ and it activated mitochondrial biogenesis to couple red cell mass to mitochondrial mass in the heart^44^. Therefore, even though we claim that upregulation of PGI_2_-dependent pathways within the coronary circulation(Fig. 4B,C) seems to represent the major compensatory mechanism in female ApoE/LDLR^−/−^ mice that contribute to their cardiac haemodynamic adaptation to exercise, many other possible mechanisms (i.e. enhanced RBC-driven ATP release, erythropoietin-mediated mechanisms, mitochondrial biogenesis in the heart, and shift in substrate metabolism in heart and skeletal muscles) could also contribute to their extraordinary exercise capacity. Moreover, higher maximal running velocity found in female ApoE/LDLR^−/−^ mice compared with age-matched C57BL6/J mice was accompanied by similar or even higher V’O_2max_ in female ApoE/LDLR^−/−^ mice (compare Fig. 1D,B,C, respectively). This could be explained by higher muscle mechanical efficiency (for overview see^45^) in female ApoE/LDLR^−/−^ mice, similar to in the trained vs. untrained humans (see^46^) and/or by higher capacity of anaerobic glycolysis and grater fatigue resistance in the locomotor muscles of the female ApoE/LDLR^−/−^ mice allowing them to maintain the exercise long after reaching their V’O_2max_. Indeed, compensatory mechanisms might operate also in the skeletal muscles of female ApoE/LDLR^−/−^ mice enabling them to run on the treadmill much better than their age-matched C57BL6/J counterparts with similar V’O_2max_.

To conclude, we claim that robust compensatory mechanisms in the hearts of female ApoE/LDLR^−/−^ mice including increased vascular responsiveness to NO and increased generation of PGI_2_ could, at least partly, explain preserved whole body V’O_2max_ as well as full cardiac adaptation to exercise of female ApoE/LDLR^−/−^ mice despite peripheral and coronary atherosclerosis progression. Our results demonstrate surprisingly efficient adaptive mechanisms in the coronary circulation in female ApoE/LDLR^−/−^ mice which when understood better, may be exploited therapeutically to limit impending impairment of cardiac output and the risk of perfusion deficit of the heart with coronary atherosclerosis.

## Methods

### Animals

ApoE/LDLR^−/−^ female mice^20^ were obtained from Jackson Lab (USA) and bred in-house. To asses exercise capacity and *in vivo* cardiac reserve, we used 6–8-month old female ApoE/LDLR^−/−^(n = 22) with advanced atherosclerosis and their age-matched wild-type female C57BL6/J (n = 26) littermates obtained from Charles River (Germany)^47^. For isolated hearts studies, 2–8 month old (2 months old, n = 79; 4 months old, n = 76; and 8 months old, n = 95) female ApoE/LDLR^−/−^ mice were used and compared to the controls (2–8 months C57BL6/J, n = 113). Mice were housed 3–5 per cage, in a temperature-controlled environment (22–25 °C) maintained on a 12 hour light/day cycle and given unlimited access to food (standard chow diet Altromin 1324 TPF (Altromin, Germany)) and water. All experimental protocols were conducted according to the Guidelines for Animal Care and Treatment of the European Union and approved by the First Local Ethical Committee on Animal Testing at the Jagiellonian University in Krakow (approvals No. 49/2009, 53/2009, 27/2014) and the Second Local Ethical Committee on Animal Testing at the Institute of Pharmacology of the Polish Academy of Sciences in Krakow (approval No. 914/2012).

### Incremental exercise for V’O_2max_ measurement

All animals were acclimatized to the closed treadmill for metabolic measurements (Columbus Instruments, Columbus, OH, USA) for three days before the experiment. Randomly selected mice from all groups (n > 9) underwent maximal incremental testing with measurement of respiratory metabolic performance according to the protocol described before^48^. Briefly, mice were run on a single-line metabolic treadmill equipped with a shock grid at 0°. The exercise protocol started at 5 m·min^−1^ and the speed was increased by 4 m·min^−1^ every 3 min until exhaustion. Data on whole body oxygen consumption (V’O_2_) were collected.

### Blood collection

Mice were anesthetized by sodium pentobarbital/pentobarbital (5:1) overdose (1 ml·kg^−1^, intraperitoneally) and the rib cage was cut along the sides to expose the heart. Blood was collected from the right ventricle into a syringe with nadroparin (10 U^·^ml^−1^) and the blood count was performed at animal blood counter Vet abc (Horiba Medical, France).

### Measurements in isolated perfused heart

The detailed technique of isolated heart perfusion to study alterations in coronary flow in mouse model according to Langendorff is described elsewhere^49^. The animals were anesthetized with mixture of ketamine (100 mg^·^kg^−1^) and xylazine (10 mg^·^kg^−1^). The hearts were precisely excised from the animal body and placed in ice-cold Krebs-Hanseleit bicarbonate buffer, where extraneous tissues were quickly removed. The aorta was attached to the prepared aortic cannula and isolated hearts were retrograde perfused with Krebs-Hanseleit buffer under 80 mmHg pressure and at 37 °C. It took approximately less than 30 seconds to mount the heart for Langendorff perfusion (hearts that required longer time for mounting were discarded from analysis). Coronary flow (CF) during the perfusion time was monitored by ultrasonic flow meter (Transit Time Flowmeter TTFM, HSE-Harvard and Transonic System Inc., USA) and flow measurements were displayed throughout the experiment at all time points. Two electrodes were placed on the right atrium of the heart model generating 400 impulses per minute. To characterize the responsiveness of coronary vessels to vasodilation several drugs were given at the volume of 10 μL each: bradykinin (Bk: NO – dependent, in 2 (n = 10), 4 (n = 10) and 8 (n = 13) months old female ApoE/LDLR^−/−^ mice and 2–4 controls (n = 16)), Acetylcholine (ACh: PGI_2_ – dependent, in 2 (n = 23), 4 (n = 16) and 8 (n = 22) months old female ApoE/LDLR^−/−^ mice and 2–4 controls(n = 25)) as well as two exogenous, endothelium-independent NO – donors with different mechanism of NO release sodium nitroprusside (SNP in 2 (n = 7), 4 (n = 8) and 8 (n = 12) months old female ApoE/LDLR^−/−^ mice and 2–4 months old controls (n = 14) and S-Nitroso-N-acetylpenicillamine (SNAP in 2 (n = 14), 4 (n = 14) and 8 (n = 15) months old female ApoE/LDLR^−/−^ mice and 2–4 months old controls (n = 16)). ACh (0.3 nmol) was injected as one bolus, whereas Bk, SNAP and SNP were injected as multiple doses (0.03; 0.1; 0.3 and 1.0 nmol). To study the contribution of NOS and cyclooxygenase (COX) to the coronary flow response separately, the nonselective NO - synthase inhibitors, NG – nitro-L-arginine methyl ester (L-NAME 500 μM in 2 (n = 6), 4 (n = 6) and 8 (n = 8) months old female ApoE/LDLR^−/−^ mice and 2–4 months old controls (n = 16)) and COX-2 selective cyclooxygenase inhibitors – Rofecoxib, 10 μM (in 2 (n = 6), 4 (n = 6) and 8 (n = 6) months old female ApoE/LDLR^−/−^ mice and 2–4 months old controls (n = 5) were used. After experiments all hearts were weighed and alterations in coronary flow were expressed in ml^·^min^·^g^−1^ of the wet ventricular weight. For determination of the concentration of the 6-keto-PGF_1α_ in effluent from the coronary vessels, samples of the effluent (500 μl) were collected and stored at −70 °C until analysed by commercially available enzyme immunoassay kits (Cayman Chemical Co, Ann Arbor, MI). 6-keto-PGF_1_α concentration was expressed in mg^·^l^−1^ (or ng^·^ml^−1^) of coronary flow (ml^·^min^−1^).

### Assessment of coronary atherosclerosis in coronary arteries

The hearts were dissected, embedded in the OCT compound (CellPath, Oxford, UK), and snap-frozen or fixed in 4% paraformaldehyde (pH 7.4), paraffin embedded and serially sectioned. Then, 10 μm thick serial cross-section slides of the heart were cut in the apex region, at the middle (at papillary muscle level) and upper part of the heart (aortic roots), to evaluate the coronary atherosclerosis in distal, intramuscular and proximal parts of coronary arteries. The sections were stained with the oil red-O for lipids detection in the atherosclerotic plaque or with the OMSB staining (Orceine with Martius, Scarlet and Blue) for histological characterisation. Photographs were acquired using the Carl Zeiss HmR monochromatic digital camera.

### Cardiac performance

Cardiac function was assessed using cardiac magnetic resonance imaging (MRI) with a 9.4T Bruker BioSpec system (Bruker, Ettlingen, Germany) equipped with BFG-113/60-S gradient system and 36 mm 1H quadrature volume resonator. Physiological parameters were monitored and gated using Model 1025 Monitoring and Gating System (SA Inc., Stony Brook, NY). Mice were measured under anesthesia maintained at 1.7% of isoflurane (Aerrane, Baxter) in a 1/2 oxygen/air mixture delivered via nose mask with monitoring of ECG, respiration and body temperature (37 °C). MRI *cine* scans were acquired with retrospectively gated IntraGate FLASH 2D sequences with the following parameters: echo time 1.49 ms, repetition time 4.3 ms, flip angle 18°, number of repetitions 250, field of view 30 × 30 mm^2^, matrix 256 × 256, slice thickness 1.0 mm and reconstructed with IntraGate 1.2.b.2 macro (Bruker) to 60 frames per cardiac cycle. LV images were analysed using the Segment package^17^ (v.1.9 R2626, Medviso AB, Sweden) and papillary muscles were included in the cavity volume. Single-slice volume was plotted against the time and used for regional cardiac functional parameters: end-diastolic/end-systolic volume (EDV/ESV), fractional area change (FAC), stroke area change (SAC) and LV filling and ejection rates (FR, ER normalized to the individual SAC and R–R values).

### Statistical analysis

Statistical analyses were performed in GraphPad Prism Software (GraphPad Software, USA) and STATISTICA 10 software (Stat-Soft Inc., USA). Results from exercise performance test, body mass and blood count were analysed with two-sided t test or non-parametric Mann-Whitney test based on the results of D’Agostino and Pearson omnibus normality test. Results from *in vivo* basic cardiac performance as well as from isolated perfused hearts were analysed by the t-test for independent variables. If parametric tests assumptions were not fulfilled (normality of distributions and homogeneity of variance), non-parametric Mann-Whitney U test or Friedman test was used. For *in vivo* cardiac reserve assessment, a repeated-measures ANOVA followed by a Tukey *post-hoc* test was used. All data were presented as mean values ± SEM.

## Additional Information

**How to cite this article**: Wojewoda, M. *et al*. Exercise capacity and cardiac hemodynamic response in female ApoE/LDLR^−/−^ mice: a paradox of preserved V’O_2max_ and exercise capacity despite coronary atherosclerosis. *Sci. Rep.* **6**, 24714; doi: 10.1038/srep24714 (2016).

## Figures and Tables

**Table 1:** Blood count.

	C57BL6/J 3m	ApoE/LDLR^−/−^ 3m	C57BL6/J 6-8m	ApoE/LDLR^−/−^ 6-8m
WBC [K^·^μl^−1^]	1.77 ± 0.28(n = 10)	3.66 ± 1.31(n = 5)	1.80 ± 0.23(n = 7)	2.48 ± 0.47(n = 5)
LYM% [%]	78.89 ± 0.5(n = 9)	71.88 ± 3.42*(n = 5)	73.74 ± 2.61# (n = 8)	73.00 ± 1.93(n = 5)
MON% [%]	5.98 ± 0.3(n = 9)	8.72 ± 0.83*(n = 5)	6.49 ± 0.53(n = 8)	7.32 ± 0.68(n = 5)
GRA% [%]	15.13 ± 0.32(n = 9)	19.26 ± 2.85(n = 5)	19.77 ± 2.57(n = 7)	19.68 ± 2.07(n = 5)
RBC [M^·^μl^−1^]	10.36 ± 0.12(n = 10)	11.51 ± 0.019***(n = 5)	9.85 ± 0.38(n = 8)	10.83 ± 0.18*^##^(n = 6)
HGB [g^·^dL^−1^]	15.08 ± 0.26(n = 10)	17.14 ± 0.14**(n = 5)	14.42 ± 0.49(n = 5)	15.28 ± 0.42^#^(n = 4)
HCT [%]	54.61 ± 0.65(n = 10)	63.48 ± 0.31**(n = 5)	52.30 ± 1.49(n = 8)	57.13 ± 1.46*^##^(n = 6)
PLT [K^·^μl^−1^]	902 ± 97.09(n = 10)	895.4 ± 35.27(n = 5)	1202 ± 67.88^#^(n = 7)	1089 ± 62.90^#^(n = 6)

WBC, white blood cells (LYM% (% of lymphocytes), MON% (% of monocytes), GRA% (% of granulocytes); RBC, red blood cells; HGB, haemoglobin; HCT, haematocrit; PLT, platelets count in young 3-month old (3m) and older 6-8-month old (6-8m) C57BL6/J and ApoE/LDLR^−/−^ mice. Statistical analysis was performed using Mann-Whitney test based on the results of D’Agostino and Pearson omnibus normality test. Data are presented as mean ± SEM. *indicates statistically significant difference between female C57BL6/J and ApoE/LDLR^−/−^ mice, ^#^indicates statistically significant age-dependent difference within the strain. ^*,#^P < 0.05, ^**,##^P < 0.01, ^***^P < 0.001.
